# The Pathogenic Potential of *Slackia exigua*: A Case Study of Bacteremia in a Patient With Oral Infection

**DOI:** 10.1155/crdi/2079663

**Published:** 2025-04-15

**Authors:** Gang Zhou, Lisa Cornelius, Dhammika H. Navarathna

**Affiliations:** ^1^Department of Pathology and Laboratory Medicine, Baylor Scott and White Medical Center, Temple, Texas, USA; ^2^Department of Medicine, Central Texas Veterans Healthcare System, Temple, Texas, USA; ^3^Department of Pathology and Laboratory Medicine Services, Central Texas Veterans Health Care System, Temple, Texas, USA

## Abstract

*Slackia exigua* is an anaerobic, Gram-positive, nonspore-forming bacterium that is typically part of the normal oral microbiota. It is generally considered biochemically inert and is often dismissed as a nonpathogenic contaminant in clinical cultures, particularly in respiratory samples. However, in this case study, we report a rare instance of *S*. *exigua* bacteremia in a patient with multiple medical comorbidities who was hospitalized after reporting to Emergency Department due to fatigue and right ear pain. The patient subsequently developed signs of persistent symptoms of septicemia, prompting an extensive investigation. Blood cultures identified *S*. *exigua* as the causative agent. This case underscores the importance of thorough culture workup and clinical correlation, especially in immunocompromised patients and patients with multiple medical comorbidities. Comprehensive dental care and the administration of targeted antibiotic therapy resulted in the patient's full recovery. This report highlights the potential pathogenic role of *S*. *exigua* in certain clinical scenarios and emphasizes the need for awareness of its possible implications in systemic infections.

## 1. Background

*Slackia exigua* is part of the normal microbiota of the human oral cavity [1]. Slackia derives from Latin ‘exigua,' meaning scanty, alluding to its sparse growth [2, 3]. *S*. *exigua* belongs to the domain Bacteria, phylum Actinomycetota, class Coriobacteriia, order Eggerthellales, family Eggerthellacaea, genus Slackia, species *Exigua*. This Gram-positive, nonspore-forming, nonmotile, obligate anaerobe. Asaccharolytic bacillus forms circular, convex, translucent colonies less than 1 mm in diameter, appearing as single rods or clumps of 0.5 μm × 1.0 μm, inert in most biochemical tests [4]. Despite its typical commensal status, *S*. *exigua* can become pathogenic under certain conditions, particularly in individuals with poor oral hygiene or underlying health conditions that compromise immune function. This bacterium is known to be associated with periodontal disease, dental abscesses, and other oral infections, and it can occasionally lead to systemic infections if not adequately managed [5, 6]. The prevalence of *S*. *exigua* as a pathogenic organism is not as high as more common oral bacteria [7]; however, its role in oral and systemic infections is increasingly recognized in clinical settings, especially among patients with complex medical histories. Poor oral health is a significant public health issue, while having comorbidities such as diabetes, chronic obstructive pulmonary disease (COPD), and substance abuse can enhance susceptibility to *S*. *exigua*.

We highlighted the importance of recognizing and managing infections caused by bacteria such as *S*. *exigua*, especially in patients with multiple comorbidities. Understanding the potential for this bacterium to cause significant health problems underscores the need for comprehensive oral hygiene and vigilant medical care in vulnerable populations.

## 2. Case Presentation

A 63-year-old male presented to the Emergency Department with fatigue and right ear pain for the last two weeks. He also reported nausea, right jaw pain, and mild shortness of breath. He was admitted for acute acetaminophen (paracetamol) toxicity after self-medicating with 1500–2000 mg up to 5 times daily for two weeks due to the ear pain. His medical history includes onychomycosis, allergic rhinitis, bilateral pseudophakia, hypertension, major depressive disorder, diabetes mellitus, obstructive sleep apnea, chronic back pain, hyperlipidemia, COPD, chronic tobacco and alcohol use (no recent history of active use), and allergies to cefaclor (resulting in face and lip swelling and pruritus) and penicillin (resulting in diffuse body rash > 40 years ago).

Physical examination was notable for poor dentition, a 3/6 systolic murmur, and a bulging, nonerythematous tympanic membrane with pain upon otoscope insertion into the right ear. He was alert and oriented, answered questions appropriately, spoke clearly, and showed no acute distress. His initial vital signs showed tachycardia (140 bpm), BP 146/94 mmHg, SpO2 97% on room air, and a temperature of 36.6°C. The patient received intravenous (IV) N-acetylcysteine (NAC; to treat acetaminophen overdose) and an IV infusion (for dehydration and nutritional deficiencies) of thiamine, folate, and multivitamin (“banana bag”). He was started on ciprofloxacin 0.3%/dexamethasome 0.1% otic suspension 4 drops 2 times a day along with vancomycin 1750 mg IV and levofloxacin 750 mg IV each as a single dose then vancomycin 1250 mg IV every 12 h and aztreonam 2 g IV every 8 h for potential dental abscess and/or ear infection and was admitted to the intensive care unit (ICU) for close monitoring and laboratory testing (WBC/DC, comprehensive metabolic panels and blood cultures). Laboratory results revealed an anion gap metabolic acidosis (bicarbonate; 10 mmol/L, pH 7.21, acetaminophen level 39 mcg/L, elevated lactic acid 7.5 mmol/L, and blood ethanol level 224 mg/dL). Diagnostic imaging at the time of ICU admission included portable chest x-ray, portable abdominal x-ray, and computed tomography (CT) mastoids and facial bones with IV contrast. Significant findings included possible periodontal disease at the left maxillary canine tooth and mild-moderate bilateral paranasal mucosal sinus disease.

**Figure 1:**
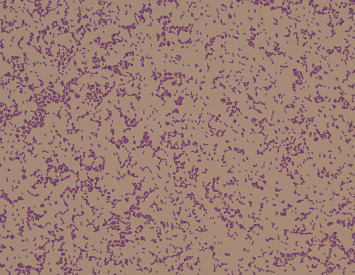
Gram stain of *Slackia exigua* pure culture showing Gram-positive rods.

After 2 days in the ICU and three doses of NAC, the patient remained hemodynamically stable, afebrile, and was transferred to the acute care unit (ACU) as microbiologic cultures (four sets of blood cultures, sputum culture, and urine culture) collected on admission due to the development of fever remained without growth. However, on the night of transfer to ACU, patient developed acute respiratory failure with narrow complex tachycardia and fever. Patient was transferred back to the ICU for closer monitoring, placed on bilevel positive airway pressure (BiPAP), and added metronidazole 500 mg IV every 8 h to the antibiotic regime. Two sets of repeat blood cultures were collected, with one anaerobic bottle growing a Gram-positive rod (Figure 1) on day five following collection, identified as *S*. *exigua* by matrix-assisted laser desorption/ionization time-of-flight mass spectrometry (MALDI-TOF-MS).

Patient was examined in a dental clinic and diagnosed with chronic periapical tooth abscess in upper left and right canine teeth and antibiotics were continued for the dental infection. Infectious diseases (IDs) specialist was consulted for the possibility that patient's fever, acute respiratory symptoms, and tachycardia may represent a reaction to antibiotics. Vancomycin, aztreonam, and metronidazole were discontinued at the 5th day and the patient was started on oral clindamycin 300 mg PO every 6 h to complete a total of 14 days. After the change in antibiotics, patient subjectively improved, defervesced, and his leukocytosis resolved. Patient underwent two dental extractions, continued to do well, and was discharged home without further incident.

## 3. Discussion

*S*. *exigua* has been associated with various infections beyond its typical oral habitat. While infections caused by *S*. *exigua* are relatively rare, several cases have been documented in the medical literature: leading to a diagnosis of perforation peritonitis highlighting the importance of advanced microbiological techniques in detecting uncommon pathogens in intra-abdominal infections [7]Few instances of monomicrobial bacteremia caused by *S*. *exigua* have been reported. One case involved community-acquired bacteremia in a patient with no prior health issues, while the second was hospital-acquired, associated with underlying conditions [8]. A 29-year-old man with a history of severe neurological deficits developed a large lung abscess due to *S*. *exigua*, suggesting its role in pulmonary infections, particularly in patients with predisposing factors [9]. In addition, an analysis of 11 clinical strains isolated from infected wound specimens identified them as *S*. *exigua* through 16S rRNA gene sequencing. This study provided the first evidence that *S*. *exigua* can be isolated from extraoral infections, expanding its known pathogenic potential beyond the oral cavity [2].*S*. *exigua* is also isolated from moderately and severely affected periodontal lesions and cariogenic oral diseases [3, 10]. A recent study found that *S*. *exigua* is more prevalent in pediatric patients compared with adults [1]. In addition, it is more common among individuals with fixed orthodontic appliances, regardless of age [11]. *S. exigua* has also been reported to cause subdural empyema (SDE) [12]. Most reported extraoral *S*. *exigua* infections were treated with favorable outcomes, except for two cases [13].

*S. exigua* is considered fastidious, requiring anaerobic bacterial growth media with Vitamin K, hemin, and L-cysteine for optimum growth and is unreactive in most conventional biochemical tests, including urease production [2, 10]. These cases illustrate the emerging recognition of *S. exigua* as a pathogen capable of causing diverse infections, including peritonitis, bacteremia, lung abscesses, and wound infections. The bacterium's fastidious nature and poor growth in laboratory settings may lead to underdiagnosis, emphasizing the need for advanced identification methods and awareness among clinicians.

*S*. *exigua* bacteremia or hematogenous disease is associated with a potential oral cavity lesion such as periapical abscess, dental caries, or periodontal disease [14, 15]. Our findings mirror these sequalae. Our lab does not provide susceptibility testing for anaerobes, which is a limitation in this study. However, the literature suggests susceptibility to most antimicrobials those include penicillin, ampicillin, ceftriaxone, imipenem, ertapenem, vancomycin, and clindamycin. It has reported resistance to sulfamethoxazole-trimethoprim [16]. Studies have shown elevated minimum inhibitory concentrations (MICs) for clindamycin, erythromycin, and trimethoprim-sulfamethoxazole against *S*.*exigua* [17] Successfully managed cases of extraoral *S*. *exigua* infections have used antibiotics such as penicillin with beta-lactamase inhibitors, ceftriaxone, and clindamycin. In our cases, due to a patient's penicillin allergy, aztreonam and vancomycin were used initially before confirming the causative organism and then transitioned to clindamycin to complete treatment. The MIC for *S*. *exigua*, measured using the E-test, showed low MICs for penicillin, ampicillin, amoxicillin-clavulanic acid, ampicillin/sulbactam piperacillin/tazobactam, ceftriaxone, imipenem, and clindamycin, making these drugs the preferred choices for treating extraoral *S*. *exigua* infections [17]. Even though CLSI or other regulatory bodies have not published breakpoints, it is useful for a microbiology lab to have the capability to test the sensitivity or resistance to commonly used antimicrobial agents against this type of anerobic pathogens.

This case highlights the broader impact of dental hygiene on systemic health. Bacteremia due to *S*. *exigua* underscores the importance of comprehensive dental care in overall health management. Similar cases reported globally [2, 8, 18, 19] demonstrate that, although rare, *S*. *exigua* can be a clinically significant pathogen and potentially fatal [12].

In our case, *S*. *exigua* contributed to the patient's right ear and jaw pain due to its presence in the oral cavity and its potential to cause localized infections, particularly in the context of poor dentition. This infection could explain patient's fatigue and nausea through systemic inflammatory responses and exacerbation of patient's chronic conditions, such as COPD and diabetes mellitus. In addition, patient's chronic alcohol use may have compromised patient's immune function, making the infection more severe and symptomatically burdensome. Given the patient's chronic health issues, careful monitoring is necessary to address both the infection and its broader impact on overall health. Chronic conditions can complicate the management of infections, making it vital to consider the patient's entire medical history when developing a treatment plan.

Furthermore, our case underscores the necessity of interdisciplinary approaches, one of the strengths in our health care system, to effectively manage *S*. *exigua* infection. Collaboration between dentists, primary care physicians, ID specialists, and laboratorians is crucial in managing complex cases where dental health intersects with other chronic health issues. Such interdisciplinary cooperation ensures a holistic approach to treatment, addressing not only the immediate infection but also the underlying health conditions that may influence patient outcomes. In addition, improving oral hygiene practices is essential to prevent recurrence and control the bacterial load in the oral cavity. This may include regular dental checkups, professional cleanings, and patient education on proper brushing and flossing techniques.

## Data Availability

All relevant deidentified data supporting the findings of this case report are included within the manuscript. No additional data are available.
